# From immune exclusion to exhaustion: tumor microenvironment drives therapy response

**DOI:** 10.3389/fimmu.2026.1785587

**Published:** 2026-04-22

**Authors:** Nemanja Maletin

**Affiliations:** 1 Faculty of Medicine, University of Novi Sad, Novi Sad, Serbia; 2 Clinic of Internal Oncology, Oncology Institute of Vojvodina, Sremska Kamenica, Serbia

**Keywords:** cancer-associated fibroblasts, hypoxia, immune exclusion, immunotherapy, metabolic reprogramming, myeloid cells, therapy resistance, tumor microenvironment

## Abstract

The tumor microenvironment (TME) is increasingly recognized as a dynamic regulator of cancer progression and therapeutic resistance. Far from being a passive scaffold, the TME comprises diverse immune and stromal components, including cancer-associated fibroblasts, myeloid-derived suppressor cells, tumor-associated macrophages, dysfunctional vasculature, and metabolic stressors, that collectively shape tumor evolution and modulate treatment response. In this review, we explore how spatial immune exclusion, immune cell dysfunction, hypoxia, and metabolic reprogramming create barriers to effective therapy, particularly in tumors refractory to immune checkpoint inhibition. We detail the molecular and cellular mechanisms by which the TME enforces immune suppression and dampens the efficacy of chemotherapy, radiotherapy, and immunotherapy. Moreover, we highlight emerging strategies to therapeutically reprogram the TME, including anti-fibrotic therapies, vascular normalization, myeloid reprogramming, metabolic modulation, and novel platforms such as oncolytic viruses, nanoparticles, and bispecific antibodies. By dissecting both established and innovative approaches, we emphasize the importance of combinatorial and context-specific interventions aimed at increasing immune accessibility and functional competence in selected contexts, thereby improving the likelihood of therapy responsiveness. A deeper understanding of the TME’s complexity offers critical opportunities to overcome resistance and improve outcomes across cancer types.

## 1 Introduction

Cancer is no longer conceptualized as a disease driven exclusively by malignant cells, but rather as a complex tissue composed of tumor cells embedded within and dynamically interacting with a heterogeneous tumor microenvironment (TME) (1–3). This paradigm shift, anticipated by Paget’s “seed and soil” hypothesis, is now firmly supported by experimental and clinical evidence demonstrating that tumor progression, metastasis, and therapeutic response are critically shaped by non-malignant components of the tumor niche (4–6). The TME is not a passive bystander but an active regulator of cancer evolution, continuously adapting to and co-evolving with malignant cells through reciprocal signaling and selective pressures (2, 7).

The TME is composed of varied immune and stromal cellular components, such as cancer-associated fibroblasts (CAFs), tumor-associated macrophages (TAMs), myeloid-derived suppressor cells (MDSCs), and regulatory T cells (Tregs), as well as cytokine mediators like interleukin-10 (IL-10) and transforming growth factor-β (TGF-β) that collectively shape immune exclusion and dysfunction (2, 3, 8). These elements form a dynamic ecosystem that governs tumor cell behavior (2, 5, 9). Importantly, substantial heterogeneity exists in the composition and spatial organization of the TME, contributing to variability in clinical outcomes (8–10).

The clinical relevance of the TME has become particularly evident in the era of targeted therapy and immune checkpoint inhibition (ICIs). Durable responses are achieved in only a subset of patients, while primary and acquired resistance remain common (11–13). TME-mediated mechanisms can modulate therapeutic response independently of tumor-intrinsic genetic alterations, underscoring limitations of biomarker strategies focused solely on cancer cell features (14–16). Instead, treatment outcomes increasingly depend on the composition, state, and spatial architecture of the TME (9, 17).

Among the most studied TME-driven determinants of therapeutic response is the immune landscape. Based on immune cell density and localization, tumors are classified into inflamed, immune-excluded, and immune-desert phenotypes (18–20). Inflamed tumors are more responsive to checkpoint blockade (19, 21), while excluded and desert types are associated with resistance (18–20). The concept of immune exclusion is central to this review but remains an evolving term across the literature. To align our usage with emerging standards, we refer to the recent modified Delphi consensus by Clifton et al. (2023), which emphasizes immune exclusion as a spatial immune topology: immune cells are present but preferentially localized to the invasive margin and/or stromal compartments with limited penetration into tumor nests, such that immune presence does not necessarily translate into effective tumor–immune engagement (22). Consistent with this framework, we distinguish three commonly used immune phenotypes: T-cell-inflamed (“hot”) tumors, characterized by abundant intratumoral T cells within tumor nests and an interferon-driven chemokine milieu; immune-excluded tumors, where immune cells accumulate in peritumoral stroma/invasive margins but fail to infiltrate tumor nests; and immune-desert tumors, defined by a paucity of T cells in both tumor nests and surrounding stroma, often reflecting impaired priming and/or poor immune recruitment (22).

Structural and cellular components of the TME enforce immune exclusion. Abnormal vasculature impairs leukocyte trafficking and promotes suppressive myeloid recruitment (1, 5). CAF-driven ECM remodeling increases stiffness and pressure, forming barriers to infiltration (2, 5). Hypoxia and metabolic stress activate immunosuppressive pathways such as VEGF and adenosine signaling (5, 9).

Even in immune-infiltrated tumors, the TME can suppress effector function. Chronic antigen exposure, inhibitory cytokines, and metabolic competition drive T-cell dysfunction, characterized by inhibitory receptor expression (14–16). TAMs, MDSCs, and Tregs reinforce suppression through IL-10, TGF-β, and other mediators, contributing to poor prognosis and therapy resistance (5, 9).

Non-immune stromal elements also shape therapy response. CAFs promote resistance via paracrine signaling and metabolic reprogramming (2, 5). Hypoxia-inducible programs support angiogenesis and stem-like traits that mediate adaptive resistance (5, 8).

Conceptually, these processes can be framed within the emerging “three Cs” model of cancer immune evasion-camouflage, coercion, and cytoprotection, which integrates how tumors avoid immune recognition, actively suppress antitumor immunity, and resist immune-mediated cytotoxicity, aligning with the continuum from immune exclusion to immune exhaustion (23).

Given the expanding body of evidence linking the TME to therapy response and resistance, there is a growing need for integrative, concept-driven syntheses that move beyond descriptive cataloging of TME components. In this review, we examine the TME as a dynamic and spatially organized regulator of therapeutic efficacy, organized around a continuum from immune exclusion to immune exhaustion. We emphasize how barriers that govern immune cell positioning (stromal/vascular/chemokine determinants) can transition into functional dysfunction under chronic antigen exposure and metabolic stress, and how immune-excluded and immune-exhausted features may coexist within the same tumor or emerge sequentially under therapeutic pressure. In addition to summarizing core mechanisms, we discuss current controversies and critical research gaps, and provide a translational synthesis linking dominant TME constraints to phenotype-matched reprogramming strategies, including clinical caveats and emerging tools (e.g., spatial and single-cell technologies) that can operationalize heterogeneity and guide biomarker-informed combination design.

This review adds an operational, phenotype-linked synthesis by mapping TME barriers to immune phenotypes and dominant resistance mechanisms, and by aligning these states with candidate therapies and biomarkers, while explicitly grading the strength of evidence across preclinical and clinical settings (**Table 1**).

**Table 1:** Integrative map of tumor immune phenotypes and actionable TME resistance nodes, with candidate therapies, biomarkers, and evidence grading.

Axis	Dominant TME barrier/node	Phenotype code*	Core resistance mechanism	Key mediators/cell types	Suggested biomarkers (examples)	Actionable strategies (examples)	Evidence code†
A	Stromal/ECM remodeling (desmoplasia)	EXC	Physical restriction of T-cell penetration into tumor nests	CAFs, collagen, hyaluronan, TGF-β, CXCL12	High TGF-β/stromal signature; FAP/CAF markers; ECM density; CD8 “at margin” spatial pattern	Anti-TGF-β ± ICI; CXCR4 blockade; selective ECM modulation	CS/P
B	Vascular dysfunction/endothelial anergy	EXC/DES	Impaired trafficking/adhesion and inefficient extravasation	VEGF, abnormal vasculature, ↓ICAM/VCAM	Angiogenic/VEGF signatures; hypoxia surrogates; endothelial adhesion markers	Anti-VEGF combinations; vascular normalization concepts	E/CS
C	Antigen presentation & priming defects	DES	Failure to generate effective T-cell priming	DC dysfunction, MHC-I loss, APM defects	MHC-I/β2M/APM status; DC density; type-I IFN signatures	STING/TLR agonists; oncolytic viruses; RT-based “*in situ* vaccination” ± ICI	E/P
D	Myeloid suppressive niche	EXC → EXH	Myeloid-mediated suppression and exclusion	TAMs, MDSCs (± TANs), IL-10, ARG1, iNOS	CD68/CD163; MDSC signatures; ARG1/iNOS; myeloid inflammation score	CSF1R-directed approaches; CD40 agonists; myeloid reprogramming + ICI (context-dependent)	E/M
E	Regulatory T-cell dominance	INF-D/EXH	Active suppression of effector T cells	FOXP3^+^ Tregs, CTLA-4, IL-10, TGF-β	FOXP3 density; IL-10/TGF-β signatures; spatial co-localization with CD8	CTLA-4 strategies; selective Treg-modulating approaches	E/CS
F	Metabolic stress (hypoxia, acidosis, nutrient competition)	EXH	Energetic collapse → loss of cytokine output and cytotoxicity	HIF-1α, lactate, adenosine (CD39/CD73→A2A), IDO-kynurenine, arginase	Hypoxia signatures; CD39/CD73; adenosine gene sets; IDO/ARG1 signatures	CD73/A2A blockade; metabolic modulation; IDO/arginase targeting (mixed clinical results)	M/P
G	Chronic antigen exposure + checkpoint network	EXH	Sustained inhibitory receptor signaling	PD-1/PD-L1, LAG-3, TIM-3, TIGIT	PD-L1 expression; IFN-γ signature; exhaustion markers (e.g., TOX); multiplex IHC	PD-1/PD-L1 inhibitors; selected dual checkpoint blockade	E

*Phenotype code definitions: DES (immune-desert): minimal/absent CD8⁺ infiltration, EXC (immune-excluded): CD8⁺ T cells restricted to stroma/invasive margin, INF-D (immune-inflamed but dysfunctional): intratumoral CD8⁺ present but suppressed, EXH (exhausted): high inhibitory receptor expression + reduced effector function. † Evidence code: E: established clinical benefit in at least some tumor settings, CS: clinical signals (early-phase, subset-specific, or context-dependent), M: mixed/negative results or inconsistent clinical translation, P: predominantly preclinical / mechanistic evidence.

## 2 Immune exclusion and spatial organization of the tumor microenvironment

Tumors vary widely in the spatial distribution of immune cells, and this organization of the TME has a profound impact on therapy response. In immunotherapy, solid tumors are often classified into three basic immunological phenotypes – T-cell-inflamed, immune-excluded, and immune-desert, based on the presence and localization of T cells in the tumor tissue (24). *Inflamed* (“hot”) tumors are characterized by a high density of CD8^+^ T cells penetrating the tumor parenchyma and stroma, reflecting an ongoing anti-tumor immune response. These T cell–rich tumors typically correlate with favorable patient outcomes and better responsiveness to therapies like ICIs (25). By contrast, *immune-excluded* tumors contain many lymphocytes at the invasive margin or in fibrous stroma, but few T cells successfully infiltrate into the tumor nests (20). Finally, *immune-desert* (immunologically “cold”) tumors lack significant T cell infiltration anywhere in the tumor or its periphery, often due to absence of T cell attraction or profound immunosuppression (16). Clinically, both excluded and desert phenotypes are associated with primary resistance to checkpoint inhibitors, since an active T cell presence is prerequisite for these therapies (16, 24). Notably, some studies suggest immune-excluded tumors can have an even poorer prognosis than completely desert tumors, underscoring the need to unravel the mechanisms behind immune exclusion (24).

### 2.1 Barriers to T cell infiltration in immune-excluded tumors

Mechanistically, the immune-excluded phenotype is driven by a TME that physically and chemically restrains T cells from reaching cancer cells. A prominent feature is an expanded stromal compartment, rich in fibroblasts and extracellular matrix (ECM), that forms a fortified barrier around tumor islets (24). CAFs in these tumors become activated (often in response to tumor-derived factors such as TGF-β and PDGF) and deposit abundant ECM components including collagen, fibronectin, and hyaluronan. The resulting fibrotic meshwork can physically trap lymphocytes in the surrounding tissue and prevent their migration into tumor nests (26, 27). Dense, aligned collagen fibers increase tissue stiffness and interstitial pressure, constraining T-cell motility, while the ECM can sequester chemokines and growth factors, disrupting the chemotactic gradients required to guide effector T cells toward malignant cells (27).

Importantly, the same desmoplastic architecture that excludes T cells also creates a transport barrier to therapy. Solid tumors such as pancreatic ductal adenocarcinoma and triple-negative breast cancer are notoriously desmoplastic, rich in collagen, hyaluronan, and contractile fibroblasts—resulting in a stiff, high-pressure interstitium that hinders therapeutic penetration (28). Under elevated solid stress and interstitial pressure, abnormal and leaky tumor vessels may collapse, producing uneven perfusion and hypoxic pockets where drugs and immune cells scarcely reach (29). Consistent with this, matrix stiffening has been linked to reduced chemotherapy uptake and efficacy in hepatocellular carcinoma (30), and drug molecules may also be sequestered or neutralized through binding to ECM components, limiting diffusion into deeper tumor regions (31). In radiotherapy, inadequate oxygenation in hypoxic niches diminishes oxygen-dependent radiosensitization, reducing radiation-induced DNA damage and increasing radioresistance (32). Collectively, these physical constraints can undermine multiple modalities—chemotherapy, radiotherapy, and immunotherapy—by limiting delivery, penetration, and intratumoral immune access (28, 32).

Beyond CAF-driven stromal barriers, myeloid programs actively reinforce immune exclusion. TAMs accumulate at the invasive margin and within stromal niches, where they sustain an immunosuppressive cytokine milieu (including TGF-β and IL-10), limit antigen presentation competence, and promote angiogenic and fibro-inflammatory circuits that collectively reduce effective T-cell trafficking and penetration (20). In parallel, tumor-associated neutrophils (TANs) can contribute to exclusion by releasing proteases and reactive mediators that remodel the ECM and by shaping chemokine landscapes that favor myeloid recruitment over cytotoxic lymphocyte entry (33). Together, CAF–myeloid crosstalk sustains a spatially segregated immune landscape in which effector T cells remain confined to peritumoral stroma rather than reaching malignant nests.

Immune and inflammatory cells within the TME actively reinforce immune exclusion and immunotherapy resistance by establishing a suppressive cytokine and chemokine milieu. TAMs, MDSCs, and regulatory T cells collectively dampen effective antigen presentation and effector priming, while sustaining inhibitory programs that restrain cytotoxic lymphocyte trafficking and function (20). TAM-derived IL-10 and TGF-β contribute to immune paralysis and tissue remodeling; notably, in a breast cancer model, macrophage-derived IL-10 inhibited dendritic cell IL-12 production, thereby blocking CD8+ T-cell activation and abrogating chemotherapy-induced antitumor immunity (34). Neutrophils and MDSCs can further amplify these circuits by providing pro-survival and pro-angiogenic signals (e.g., VEGF, CXCL8) and by supporting tissue repair after therapy-induced damage, which indirectly preserves a hostile niche for T-cell entry (35). Consistent with this, stromal–myeloid crosstalk can recruit and sustain suppressive myeloid populations, which in turn release mitogens and proteases that maintain exclusion and facilitate tumor persistence under immune pressure (36). In aggressive settings such as hepatocellular carcinoma, targeted therapy can trigger neutrophil accumulation and subsequent recruitment of TAMs and T_regs, collectively fostering an environment permissive to tumor regrowth and resistant to immune-mediated clearance (37).

In addition to forming a physical barrier, CAFs actively sculpt an immunosuppressive microenvironment through secreted factors. They produce chemokines and cytokines (including stromal cell–derived factor 1, SDF-1/CXCL12; vascular endothelial growth factor, VEGF; and TGF-β) that can misdirect, exclude, or inhibit immune cells. For example, CAF-derived CXCL12 can act as a decoy that limits T-cell access to tumor cells, while TGF-β further amplifies fibroblast activation and reinforces lymphocyte exclusion. Consistent with this framework, gene expression analyses show that immune-excluded tumors are enriched in stromal and TGF-β response signatures, whereas inflamed tumors display interferon signaling and T cell–attracting chemokines (38). Therapeutically, stromal reprogramming strategies can mitigate immune exclusion; notably, combined TGF-β pathway inhibition and anti–PD-(L)1 therapy has been shown to reduce fibrotic barriers and increase intratumoral T-cell infiltration in preclinical models, although clinical efficacy appears context-dependent and remains under active evaluation (39, 40). More broadly, efforts to normalize tumor vasculature and ECM (including anti-angiogenic and anti-fibrotic strategies) can improve transport and create conditions more permissive to immune infiltration, although clinical benefit remains context-dependent and often transient (20).

### 2.2 Vascular and chemokine determinants of immune cell positioning

A second major driver of immune exclusion is the abnormal tumor vasculature. The neovasculature in tumors is typically disorganized and poorly functional, leading to regions of hypoxia and acidosis that are hostile to effector T cells (39, 40). Importantly, these conditions are not merely downstream consequences of poor perfusion, but can also reinforce endothelial anergy and dampen T cell–attracting chemokine programs, thereby stabilizing immune exclusion. Tumor blood vessels also actively limit lymphocyte entry by disrupting both key steps of recruitment: (i) endothelial adhesion and extravasation, and (ii) chemokine-guided navigation into the tissue. Tumor-associated endothelial cells downregulate leukocyte adhesion molecules such as E-selectin, ICAM-1 and VCAM-1, resulting in impaired docking and transmigration of circulating T cells into the tumor parenchyma (39). In parallel, the same dysfunctional endothelium and perivascular niche may suppress T cell–attracting chemokine programs (e.g., CXCL9/10/11- and CCL5-driven gradients), weakening the directional cues needed for T cells to penetrate beyond peritumoral stroma even when they reach the vessel wall (39).

Mechanistically, this endothelial “anergy” is driven by chronic exposure to angiogenic and immunosuppressive mediators (notably VEGF, TGF-β, prostaglandin E₂ and IL-6), which blunts inflammatory activation and reduces NF-κB–dependent induction of adhesion molecules in response to cytokines such as TNF-α and IFN-γ (41). Hypoxia further reinforces this phenotype by sustaining pro-angiogenic signaling and stabilizing suppressive programs in endothelial and perivascular cells (39, 40). As a consequence, even if T cells are present in the bloodstream, they fail to extravasate efficiently and lack appropriate chemotactic guidance, yielding the characteristic spatial segregation of immune-excluded tumors. Moreover, the aberrant endothelium can function as an immunological “checkpoint” itself: it preferentially permits trafficking of immunosuppressive myeloid cells (e.g., MDSCs) rather than cytotoxic T lymphocytes, and it can express inhibitory ligands such as PD-L1 while releasing soluble factors that dampen T cell activity. This endothelial barrier further skews the immune infiltrate toward tumor-promoting cells and away from tumor-attacking lymphocytes.

### 2.3 Immune phenotypes and response to therapy

The distinction between *“hot”* and *“cold”* tumors is not merely academic, but has direct therapeutic relevance. Inflamed tumors, with their pre-existing TILs, are precisely the settings where ICIs like anti-PD-1 or anti-CTLA-4 tend to unleash a robust anti-tumor effect (25, 42). In these tumors, effector T cells are already present and poised for action once inhibitory brakes are removed. In addition to immune contexture, tumor-intrinsic antigenicity is a key determinant of checkpoint inhibitor benefit. Across multiple tumor types, patients with a high tumor mutational burden (TMB) and a higher predicted neoantigen load tend to achieve higher response rates and more durable benefit from PD-1/PD-L1 and/or CTLA-4 blockade, consistent with the notion that increased numbers of non-self neoepitopes expand the pool of tumor-reactive T-cell clones that can be reinvigorated by ICI therapy (43, 44). Conversely, low-TMB tumors generally generate fewer neoantigens and are less likely to mount effective pre-existing antitumor immunity, contributing to primary resistance. However, TMB is not universally predictive, and its clinical utility depends on tumor type and the broader immune context.

In contrast, immune-excluded and -desert tumors are often intrinsically resistant to ICIs, since T cells either cannot reach the tumor cells or are entirely absent (20, 45). As a result, there are simply no anti-tumor T cells to invigorate with checkpoint blockade in a cold tumor (20, 45). This explains why patients with T cell–poor tumors have low response rates and why their overall prognosis on immunotherapy is poor. For example, extensive analyses of clinical samples have shown that patients with T cell–excluded tumors derive significantly less benefit from PD-1/PD-L1 inhibitors, and in some cohorts these patients had shorter survival than even those with completely immune-desert tumors (26). The spatial immune phenotype is therefore a critical biomarker of immunotherapy outcome.

Encouragingly, recognizing the TME’s role in immune exclusion has prompted new combination therapies aimed at transforming cold/excluded tumors into inflamed ones. Approaches such as CAF reprogramming, TGF-β inhibition, ECM modulation, and vascular normalization are being explored to reduce stromal and vascular constraints; however, despite robust preclinical rationale, the clinical translation of anti-angiogenic strategies to ‘open’ the TME has been heterogeneous and remains largely context- and regimen-dependent. This underscores the need for biomarker-guided patient selection, dose/schedule optimization (including the concept of a transient ‘normalization window’), and rational combination design rather than assuming broad clinical efficacy (46, 47). By normalizing blood vessels and reducing fibrosis, these strategies can facilitate T cell infiltration and boost the efficacy of ICIs. In summary, the spatial organization of the TME, whether permissive or prohibitive for immune cell entry, is a key determinant of therapeutic response. T-cell-inflamed tumors are primed for immune-mediated clearance, whereas immune-excluded and -desert tumors mount a formidable defense via stromal, vascular, and molecular mechanisms to exclude T cells and resist immunotherapy (46). At the molecular level, immune exclusion is sustained by suppressive cytokine programs (notably TGF-β and IL-10), CAF-driven chemokine “misdirection” (e.g., CXCL12) together with attenuation of T cell–attracting chemokine gradients (CXCL9/10/11), and endothelial/metabolic checkpoints that impair recruitment and function (VEGF-driven endothelial anergy with reduced E-selectin/ICAM-1/VCAM-1, as well as hypoxia-linked adenosine accumulation via CD39/CD73 signaling) (38–40). Understanding and modulating these mechanisms of immune exclusion is pivotal for overcoming resistance and improving cancer immunotherapy outcomes (24). Importantly, immune exclusion is not always a terminal endpoint. Under sustained myeloid-dominant suppression and metabolic checkpoint pressure (e.g., hypoxia–adenosine signaling), residual or newly recruited T cells may transition from spatially restricted states toward dysfunctional/exhausted programs under chronic antigen exposure, providing a mechanistic bridge along the EXC to EXH continuum (**Table 1**) (38–40).

## 3 Immune dysfunction and exhaustion in the tumor microenvironment

Tumors can actively subvert host immunity by shaping the tumor microenvironment into an immunosuppressive niche in which infiltrating effector T cells progressively become dysfunctional or exhausted. Consequently, even T cell–inflamed (“hot”) tumors may evade therapy because antitumor T cells are present but functionally constrained by local suppressive conditions.

### 3.1 T-cell exhaustion and inhibitory signaling

Chronic antigen exposure in the tumor, combined with continuous inhibitory signals, drives T cell exhaustion. Exhausted T cells (Tex) were first identified in chronic viral infections and are now recognized in many cancers (48). In tumors, persistent T-cell receptor stimulation without relief leads to a distinct differentiation state marked by loss of effector functions (e.g. reduced cytokine secretion and cytotoxicity) and overexpression of inhibitory receptors (48–50). These T cells upregulate checkpoints such as PD-1, CTLA-4, TIM-3, and LAG-3 – often co-expressing several at once – which actively dampen their responsiveness (51). Phenotypically, Tex cannot proliferate or produce IL-2 and IFN-γ effectively, and they express transcriptional profiles distinct from functional effector or memory T cells (51). The presence of such Tex in the TME is associated with poor clinical outcomes across multiple cancers (48, 52), as the immune system is essentially present but ineffective.

A major driver of T cell dysfunction in tumors is inhibitory signaling through checkpoint receptors. For example, engagement of PD-1 on T cells by PD-L1/PD-L2 in the TME delivers a “stop” signal that blunts kinases needed for T cell activation. Similarly, CTLA-4 on T cells competes with CD28 for B7 ligands on antigen-presenting cells, blocking co-stimulation. The net effect is a profound functional brake on T cells. Tex typically display high PD-1 alongside other checkpoints like TIM-3 and LAG-3 (53–55). These redundant inhibitory pathways reinforce dysfunction, indeed, PD-1 signaling was shown to reduce T-cell metabolic fitness (downregulating the glucose transporter GLUT1 and glycolytic capacity) (56). The prevalence of multiple checkpoints on Tex is a hallmark of their stable dysfunction. Notably, these inhibitory mechanisms have physiologic roles (e.g. preventing autoimmunity during chronic immune activation), but tumors hijack them to protect themselves. In the TME, tumor and stromal cells often overexpress checkpoint ligands (PD-L1, PD-L2, B7-family molecules, etc.), relentlessly engaging T cell inhibitory receptors and maintaining T cell unresponsiveness (57).

To clarify terminology and reduce overlap between closely related states, we summarize key distinguishing features of functional effector, early dysfunctional (potentially reversible), and exhausted CD8^+^ T-cell states in the tumor microenvironment (**Table 2**).

**Table 2:** Distinguishing functional effector, dysfunctional, and exhausted CD8^+^ T-cell states in the tumor microenvironment.

Feature	Functional effector CD8^+^ T cells	Dysfunctional/early dysfunctional (potentially reversible)	Exhausted T cells (Tex; advanced/terminal)
Spatial context in TME	Typically present in inflamed tumors; effective tumor contact	Often present but functionally constrained by suppressive niche	Present in chronically inflamed TME with sustained suppression
Cytokine production	High IFN-γ, TNF-α, IL-2	Reduced (often selective loss of IL-2 first)	Markedly reduced; blunted polyfunctionality
Cytotoxicity	High perforin/granzyme; effective killing	Reduced killing capacity	Low killing capacity despite presence of CD8^+^ cells
Proliferation	High	Variable/moderate	Low (especially terminal Tex)
Inhibitory receptors (IRs)	Low/basal	PD-1↑ (± TIM-3/LAG-3)	PD-1↑↑ with co-expression of TIM-3/LAG-3/TIGIT (often multi-IR)
Transcriptional program	Effector program dominant	Mixed program; stress/adaptation signals	Exhaustion program dominant (e.g., TOX-high; epigenetic fixation)
Metabolic fitness	Preserved glucose uptake and mitochondrial function	Metabolic stress; competition for nutrients; early mitochondrial strain	Severe metabolic constraints (hypoxia, adenosine, lactate); mitochondrial dysfunction
Key extrinsic drivers	Adequate priming; supportive cytokine milieu	TGF-β/IL-10, myeloid suppression, early checkpoint signaling	Chronic antigen exposure + sustained checkpoint signaling; adenosine axis; persistent hypoxia/acidosis
Reversibility	Not applicable	Often partially reversible	Frequently poorly reversible (especially terminal Tex)
Predicted response to ICI	Not applicable/already active	Often more responsive	Variable; terminal Tex tends to respond less

### 3.2 Cellular composition of the TME

The cellular composition of the TME is another critical determinant of T cell dysfunction. Tumors are infiltrated by various suppressive immune cells that dampen T cell responses despite the presence of antigens.

#### 3.2.1 Suppressive immune cells

Among the most prominent are regulatory T cells (Tregs). These FOXP3^+^ CD4 Tregs are normally vital for self-tolerance, but in tumors they accumulate in abnormal numbers and skew the balance toward immunosuppression. Tregs in the TME secrete high levels of IL-10 and TGF-β (potent immunosuppressive cytokines) and express CTLA-4, through which they engage and downregulate costimulatory molecules on dendritic cells (DCs) (3). They also consume IL-2 (via their high-affinity IL-2 receptor CD25), depriving conventional T cells of this growth factor. The combined effect is that Tregs prevent the activation and expansion of effector T cells, essentially creating an immune privileged niche for the tumor (58, 59). Clinically, a high density of Tregs in tumors correlates with worse prognosis in many cancer types (e.g. ovarian, breast, pancreatic cancers), underlining their role in blunting anti-tumor immunity. Tumors actively recruit Tregs via CCL22 and other chemokines, as evidenced in ovarian carcinoma where Treg recruitment fosters immune privilege and reduces patient survival (26). Thus, Tregs are key enforcers of immune dysfunction in the TME.

MDSCs represent a major immunosuppressive population in tumors. These pathologically activated immature myeloid cells expand in cancer and potently suppress T-cell responses through multiple mechanisms (60). MDSCs commonly express arginase-1 (ARG1) and inducible nitric oxide synthase (NOS2), depleting L-arginine and generating nitric oxide (NO), which together impair T-cell proliferation and signaling (including reduced TCR ζ-chain expression and NO-mediated inhibition of signaling molecules) (61, 62). By combining nutrient depletion with oxidative/nitrosative stress, MDSCs establish a hostile milieu for effector T cells (63). They further suppress immunity by producing IL-10 and TGF-β and by expressing PD-L1, enabling contact-dependent inhibition (64). Importantly, MDSCs can amplify suppression via crosstalk, promoting Treg expansion and skewing macrophages toward an M2/TAM-like phenotype (65). Clinically, elevated MDSC levels correlate with poorer therapeutic responses (including to checkpoint inhibitors and chemotherapy), consistent with a T cell–excluded or suppressed TME state (66, 67).

TAMs are often the most abundant immune infiltrate and a cornerstone of immune evasion. They are frequently polarized toward an M2-like program linked to tissue remodeling and immunoregulation, characterized by IL-10 high/IL-12 low profiles and expression of markers such as the mannose receptor (68, 69). TAMs suppress T-cell function and support tumor progression via immunoregulatory cytokines (IL-10, TGF-β), chemokines that recruit additional suppressive cells (Tregs/MDSCs), and angiogenic factors (VEGF, bFGF) that can indirectly limit immune infiltration (70). They also reinforce metabolic and enzymatic suppression (e.g., arginase, IDO, competition for glucose, particularly under hypoxia, and adenosine generation via CD39/CD73) (71), and they remodel tissue architecture by promoting fibrosis and extracellular matrix deposition, creating barriers to T-cell entry and drug delivery (72). Clinically, high TAM density is generally associated with adverse outcomes, and in several tumor types macrophages can constitute a substantial fraction of the tumor mass, with higher TAM burden correlating with shorter survival (3). Overall, MDSCs and TAMs act as key orchestrators of a suppressive TME that constrains effective antitumor T-cell immunity and contributes to therapy resistance (73).

While the review emphasizes T-cell–centric mechanisms, innate immune components critically shape whether effective antitumor T-cell responses can be generated and sustained. DCs (especially cross-presenting cDC1) are central to priming tumor-reactive CD8+ T cells, yet are often numerically or functionally compromised in tumors by hypoxia, acidosis, and suppressive myeloid mediators; spatially restricted DC dysfunction can therefore create a “priming bottleneck” even in antigenic tumors (74). Natural killer (NK) cells provide complementary tumor surveillance, can eliminate antigen-presentation–deficient tumor variants, and produce cytokines that support DC–T-cell crosstalk; however, NK cell infiltration and cytotoxicity are frequently constrained by metabolic stress and inhibitory ligands within the TME (75, 76). These innate programs are increasingly recognized as actionable determinants of immune phenotypes and treatment response, particularly in tumors with low T-cell priming or defective antigen presentation.

Beyond checkpoints, metabolic reprogramming approaches are being explored to restore T-cell activity in suppressive TMEs, particularly by targeting adenosine signaling and nutrient-depletion pathways (49, 50, 77). Inhibiting the IDO–kynurenine axis has shown mixed clinical results to date, yet remains an area of active investigation (78). Additional strategies include counteracting arginine depletion (e.g., arginase inhibition) and broader efforts to improve T-cell metabolic fitness, although much of this remains preclinical (79, 80).

Beyond checkpoint blockade, strategies that deplete or reprogram suppressive TME populations (Tregs, MDSCs, and TAMs) are being developed to relieve dominant inhibitory circuits and enhance effector function (80–85). Representative approaches include limiting suppressive-cell trafficking, inducing differentiation programs, or repolarizing myeloid cells toward pro-inflammatory states, often to improve responses to ICIs. Vascular normalization may further support infiltration by alleviating delivery constraints and hypoxia-associated suppression (81–85).

Selected ongoing and completed clinical trials targeting immune exclusion and dominant immunosuppressive axes (e.g., TGF-β, adenosine/CD73–A2A, CXCR4–CXCL12) are summarized in **Supplementary Table 1** (trial identifiers provided).

## 4 Metabolic and hypoxic remodeling of the TME: mechanisms shaping immune function

Tumor tissues typically outgrow their blood supply, leading to regions of hypoxia (low oxygen) and nutrient deprivation. Unlike normal organs, tumor vasculature is chaotic and poorly functional vessels are leaky, tortuous, and unevenly distributed (86). This aberrant vascular architecture, together with the high oxygen and glucose consumption of rapidly proliferating cancer cells, creates heterogeneous perfusion deficits and chronic hypoxia in the tumor core and in poorly perfused niches. Hypoxia is therefore not a rare “late event,” but a recurring and spatially dynamic hallmark of many solid tumors, emerging early during malignant expansion and persisting as tumors remodel their stroma and vasculature (86).

Hypoxic zones trigger broad changes in gene expression that enable tumor cell survival and resistance to apoptosis (39, 87). A central mediator is stabilization of hypoxia-inducible factor-1 (HIF-1), a transcription factor that activates dozens of adaptive genes supporting angiogenesis, survival, and metabolic rewiring. HIF-1 upregulates vascular endothelial growth factor (VEGF), which stimulates new blood vessel growth in an attempt to restore oxygen delivery (88). However, the resulting neovasculature is frequently abnormal and inefficient, so oxygen supply remains insufficient—creating a self-reinforcing cycle in which hypoxia induces angiogenesis, yet angiogenesis fails to fully relieve hypoxia (86). Tumor expansion also exerts physical pressure that can collapse vessels, further exacerbating hypoxia and elevating interstitial fluid pressure. Dvorak and colleagues describe how compressive stress in tumors can block blood flow, inducing hypoxia and VEGF expression in surrounding tissue (89). Collectively, these processes explain why the TME is often characterized by patchy oxygen deprivation, fluctuating perfusion, and steep gradients of oxygen, metabolites, and pH.

Tumor hypoxia not only promotes angiogenesis but also induces broad cellular adaptations, largely through HIF-1/2–driven transcriptional programs, including altered gene expression, suppression of apoptosis, activation of autophagy, promotion of epithelial–mesenchymal transition (EMT), malignant progression and metastasis, and metabolic reprogramming (90, 91).

### 4.1 Hypoxia-driven immunosuppression and the HIF-1 program

The metabolic environment of tumors, largely shaped by poor perfusion and deregulated cancer cell metabolism, imposes additional therapy resistance across modalities. Chronic hypoxia triggers adaptive responses that make cancer cells less susceptible to both drugs and radiation. Hypoxia-inducible factor 1α (HIF-1α), stabilized in low oxygen conditions, upregulates genes that help cells survive stress, including drug efflux transporters and anti-apoptotic proteins (92). For example, HIF-1–driven expression of P-glycoprotein (MDR1) can pump out chemotherapeutics, conferring multidrug resistance in colon cancer (92). Notably, reversing hypoxia (or blocking HIF-1 activity) can resensitize tumors to chemotherapy, underscoring how central oxygen deprivation is to chemoresistance (93). Hypoxia also compromises radiotherapy, as oxygen is required to “fix” DNA damage induced by ionizing radiation; accordingly, heavily hypoxic tumors such as pancreatic cancer and glioblastoma tend to respond poorly to radiotherapy unless reoxygenation strategies are employed.

Tumor metabolic reprogramming further generates a harsh acidic microenvironment that thwarts therapy (94). Tumor cells produce excess lactic acid (via glycolysis) and hydrogen ions, lowering extracellular pH in poorly perfused regions. This acidity can directly impair certain drugs, for instance, weakly basic chemotherapeutics may become protonated and trapped in acidic compartments, reducing their bioavailability to tumor cells. Acidic pH also promotes autophagy as a survival mechanism; indeed, chronic acidosis has been linked to upregulation of autophagy-related genes (e.g., ATG5, BCL-2), enabling tumor cells to withstand therapy-induced stress (95). Moreover, an acidic, nutrient-depleted TME debilitates immune effector cells, T lymphocytes and NK cells function sub-optimally in low pH and hypoxic conditions, diminishing the effectiveness of immunotherapies. For example, accumulated lactic acid and adenosine in hypoxic TMEs can drive immunosuppressive polarization programs affecting DCs and T cells (96). Collectively, the hypoxic, acidic, nutrient-poor milieu acts as a selective filter against therapies, enabling survival of cancer cells with robust stress-response pathways under treatment pressure.

Beyond these broad stress adaptations, hypoxia has profound consequences in the TME by activating immunosuppressive and pro-survival pathways. A major effect is HIF-1 signaling, which not only promotes angiogenesis but also reshapes immune modulators and stromal behavior. HIF-1 can induce tumor and stromal cells to express immunosuppressive mediators; notably, it upregulates ectoenzymes CD39 and CD73, enabling the generation of extracellular adenosine, a potent inhibitory metabolite (91). In the oxygen-starved TME, ATP released from dying or stressed cells is degraded to adenosine by CD39/CD73, and adenosine can accumulate to high levels (72). Adenosine engagement of A2A receptors on immune cells (T cells, NK cells, and others) triggers cyclic AMP signaling that suppresses cytotoxic function and curtails antitumor immunity (31). Mechanistically, A2A receptor signaling elevates intracellular cAMP and dampens effector programs, reducing IFN-γ production and cytotoxic mediator output (97). In parallel, inhibitory checkpoint signaling (notably PD-1) enforces a metabolically constrained state by suppressing glycolysis and promoting lipid/FAO programs, thereby limiting the capacity of effector T cells to sustain cytokine production (e.g., IFN-γ) and cytotoxic molecules such as perforin and granzymes in the nutrient-poor TME (98). Hatfield et al. (2016) describe this “hypoxia–adenosine axis” as a major barrier to effective immune control: hypoxic, adenosine-rich microenvironments can paralyze T and NK cells unless the pathway is disrupted (91). Accordingly, strategies to oxygenate tumors or inhibit adenosine signaling (outlined below) are mechanistically attractive because they target a central metabolic-immunologic checkpoint rather than a single cytokine.

Hypoxia also skews immune cell recruitment and polarization. TAMs tend to accumulate in hypoxic zones, guided by hypoxia-inducible chemoattractants including VEGF and endothelins. Within low oxygen niches, TAMs frequently adopt M2-like, pro-tumoral programs. In the hypoxic milieu, they upregulate reactive oxygen species (ROS) and enzymes such as inducible nitric oxide synthase, which can react to form peroxynitrite—molecules capable of inducing T-cell dysfunction and broader tissue damage (99). Whiteside’s review emphasizes that hypoxia-resident phagocytes (macrophages, neutrophils) can produce abundant ROS, activate NF-κB and related pathways, and thereby blunt effective T-cell responses. DCs are likewise impaired: the hypoxic, acidic conditions in tumors reduce DC maturation and antigen-presenting capacity, undermining T-cell priming (100, 101). Furthermore, hypoxia can induce checkpoint molecules; for example, HIF-1 can elevate PD-L1 expression on tumor cells, directly inhibiting T-cell activity (102, 103). Together, these effects illustrate how hypoxia remodels the TME into a tolerogenic landscape: high VEGF and adenosine levels, suppressive myeloid accumulation, increased checkpoint pressure, and shifts in cytokine profiles (e.g., elevated IL-10 and TGF-β), all converging toward immune escape (104).

### 4.2 Metabolic reprogramming, nutrient competition, and immune paralysis

Compounding the effects of hypoxia, tumor cells undergo profound metabolic reprogramming that further shapes an immunosuppressive TME. A central feature is the Warburg effect, in which tumors preferentially rely on aerobic glycolysis, resulting in excessive glucose consumption and accumulation of lactate and protons (36). This metabolic phenotype creates glucose depletion and acidification of the TME—two stressors that directly impair antitumor immunity. Tumor glucose uptake can metabolically starve tumor-infiltrating T cells, suppressing their glycolytic capacity and IFN-γ production, ultimately promoting tumor progression (105). Under glucose-limited conditions, effector T cells cannot sustain the energetic and biosynthetic programs required for proliferation and cytotoxic function, and they activate stress-response pathways such as AMPK and GCN2. In practice, this means that even when immune cells are present in the tumor, their functional output can be throttled by resource scarcity, turning infiltration into ineffective “bystander inflammation” rather than productive tumor control.

Beyond glucose, tumors also deplete essential amino acids required for T-cell function. A prominent example is tryptophan, degraded by indoleamine-2,3-dioxygenase (IDO) expressed by tumor and myeloid cells (106). IDO-mediated tryptophan depletion induces T-cell anergy and abortive proliferation, triggers GCN2 activation, and promotes regulatory T-cell differentiation (107). Concurrently, accumulation of kynurenine suppresses immunity via activation of the aryl hydrocarbon receptor, reinforcing immunosuppressive phenotypes in T cells and myeloid populations. Similar metabolic suppression occurs through arginine depletion by arginase-expressing myeloid cells, which impairs T-cell receptor signaling and reduces effector function (108, 109). Together, these nutrient-deprivation mechanisms establish a metabolic “checkpoint layer” in the TME that can persist even when classical checkpoints are therapeutically blocked. Glutamine metabolism also contributes to cancer immune evasion. Many tumors exhibit “glutamine addiction,” creating a nutrient tug-of-war that deprives effector lymphocytes of glutamine required for activation and proliferation, while glutamine catabolism in the TME supports suppressive myeloid programs and dampens antitumor immunity. Accordingly, glutamine blockade can reprogram the TME and enhance antitumor immune responses in preclinical models, although clinical translation remains under active investigation (110, 111).

### 4.3 Lactate, acidity, and epigenetic reinforcement of immunosuppression

The metabolic end-products of tumor glycolysis further contribute to immune evasion. Lactic acid is exported into the extracellular space, causing lactate accumulation and extracellular acidification; interstitial pH may drop to 6.5–6.8. Acidic conditions directly impair T-cell and NK-cell function, reducing cytotoxicity and decreasing production of effector molecules such as IFN-γ and perforin. Importantly, lactate is not merely a waste product: it also acts as an active immunomodulatory signal (112). Elevated lactate promotes M2-like macrophage polarization and increases secretion of matrix metalloproteinases, facilitating tumor invasion (113). Lactate can additionally serve as a metabolic fuel for TAMs, reinforcing immunosuppressive behavior under conditions in which effector lymphocytes are metabolically disadvantaged (114).

Recent studies further highlight that lactate can induce epigenetic modifications, including histone lactylation, which upregulates immunosuppressive genes such as arginase-1 and IL-10 in macrophages and increases expression of exhaustion markers, including PD-1, in T cells (115, 116). These observations are particularly relevant because they suggest that metabolic stress can stabilize suppressive immune states beyond transient signaling, embedding them within transcriptional and epigenetic programs. Consistent with this, tumor regions characterized by high lactate and low pH are often associated with reduced immune infiltration and poor prognosis (117). In parallel, oxidative and nitrosative stress, driven by excessive production of reactive oxygen and nitrogen species by tumor and myeloid cells, further suppresses immunity by damaging T cells and disrupting chemokine-mediated trafficking (118).

### 4.4 Consequences for therapy response and resistance across modalities

These hypoxic and metabolic features of the TME directly affect therapy response, fostering resistance to both traditional and newer treatments. In radiotherapy, oxygen is a critical radiosensitizer because oxygen fixes radiation-induced DNA damage into lethal lesions. Hypoxic tumor cells, lacking sufficient O₂, are markedly more resistant to radiation; historically, hypoxic cells may require ~2–3 times higher radiation dose to achieve comparable cell kill as normoxic cells (119). HIF-1 activation also triggers DNA damage response pathways and may shift cells into radiotherapy-insensitive cell cycle states, further contributing to radioresistance (120). Accordingly, tumor hypoxia is correlated with poor radiotherapy outcomes in several malignancies, including head-and-neck carcinoma and sarcomas (121).

Many chemotherapeutics are also less effective under hypoxic and acidic conditions: poor perfusion limits drug delivery to tumor cores, oxygen-dependent drug chemistry can be compromised, and acidic pH can reduce uptake of weakly basic drugs while degrading certain cytotoxics (119). Beyond physicochemical barriers, the immunosuppressive TME sabotages modern immunotherapy. ICIs require functionally competent T cells; however, in hypoxic, nutrient-poor, lactate-rich tumors, T cells may remain metabolically constrained even if checkpoint signaling is therapeutically relieved. Indeed, ICI non-responders frequently exhibit features of a metabolically hostile TME, and tumors with low glucose/high lactate often resist PD-1 blockade (122). Hypoxia-induced PD-L1 expression on tumor cells can further sustain inhibitory pressure, limiting durable immune activation (86). Similarly, adoptive cellular therapies (e.g., CAR T-cell or TIL therapy) may fail when infused T cells encounter an “acidic, adenosine-rich” microenvironment and rapidly lose functionality. Overall, metabolic and hypoxic remodeling represents a fundamental mode of therapy resistance, shielding cancer cells from radiation and drugs while erecting a metabolic barricade against immune-mediated killing (123).

### 4.5 Therapeutic strategies to target hypoxia and metabolism

Given the central role of hypoxia and metabolic reprogramming in therapy resistance, multiple strategies aim to target or reverse these TME features. One major approach focuses on tumor vasculature and hypoxia. Anti-angiogenic therapies such as bevacizumab were initially designed to starve tumors, but are now recognized to transiently normalize abnormal blood vessels (124). By pruning dysfunctional vasculature and tightening endothelial junctions, anti-VEGF treatment can temporarily improve oxygenation and drug delivery before vessel regression (125). Improved oxygen supply can enhance radiosensitivity and may attenuate HIF-1α–driven immunosuppressive pathways, although careful timing is required to exploit the normalization window.

Additional approaches aim to directly overcome hypoxia. These include hyperoxygenation strategies such as carbogen breathing or hyperbaric oxygen during radiotherapy, as well as emerging technologies like oxygen microbubbles, oxygen carriers, and hypoxia-activated prodrugs (e.g., tirapazamine, evofosfamide) that selectively target hypoxic tumor cells (126). Targeting the adenosine pathway is another key strategy. Small-molecule A2A receptor antagonists and monoclonal antibodies against CD73 are currently being tested, often in combination with ICIs (127). Preclinical studies indicate that blocking adenosine signaling can reinvigorate T cells and NK cells, thereby enhancing antitumor immunity (128).

Metabolic checkpoint inhibition is also under active investigation. IDO1 inhibitors were developed to prevent tryptophan depletion and kynurenine accumulation, although clinical results have been mixed, motivating ongoing efforts to refine patient selection and combination strategies. In parallel, arginase inhibitors are being tested to restore arginine availability and T-cell proliferation (129). Additional approaches aim to reprogram tumor metabolism directly, including inhibitors of glucose transport, glycolysis, lactate production, and lactate export (e.g., GLUT1, hexokinase, LDH, and MCT inhibitors), with the goal of reducing lactate accumulation and acidosis. While preclinical data are encouraging, clinical translation has been challenging due to toxicity and metabolic complexity (50, 130). An alternative strategy involves buffering tumor acidity (e.g., systemic buffers or proton pump inhibitors), which has shown early promise in enhancing T-cell infiltration and adoptive cell therapy efficacy (131).

Ultimately, combinatorial approaches are likely required. Because hypoxia and metabolism form interconnected barriers, rational combinations, such as adenosine blockade with lactate inhibition alongside ICIs, may yield synergistic benefits. These approaches aim to increase immune accessibility and intratumoral effector function in otherwise resistant, immune-excluded or ‘cold’ tumors and, in selected settings, to shift them toward more inflamed phenotypes with improved therapy responsiveness. Targeting both cancer cells and their microenvironment thus represents a critical frontier in overcoming tumor-mediated therapy resistance.

Selected clinical trials supporting angiogenesis–hypoxia–metabolic targeting strategies (trial identifiers provided where applicable) are listed in **Supplementary Table 2**.

## 5 TME-mediated resistance across therapy modalities

The TME comprises a dense network of cancer cells surrounded by non-malignant support cells (fibroblasts, immune cells, endothelial cells, etc.), abnormal vasculature, and ECM deposits. These components create physical barriers (fibrotic stroma, high interstitial pressure, irregular blood flow) and chemical gradients (hypoxia, acidity) that impede the efficacy of diverse cancer therapies (132, 133). Notably, tumors often behave as “wounds that do not heal,” co-opting normal wound-healing processes to constantly remodel the stroma in a pro-tumorigenic manner. Such an abnormal microenvironment can fuel tumor progression and broadly promote resistance to chemotherapy, targeted therapy, radiotherapy, and immunotherapy (134).

### 5.1 Physical barriers to drug delivery and infiltration

As discussed above, desmoplasia and abnormal vasculature create transport barriers that limit both immune infiltration and drug delivery; here we focus on additional cellular and paracrine mechanisms by which the TME actively drives resistance across therapy modalities.

### 5.2 Stromal programs (CAFs and ECM): paracrine protection and survival niches

Beyond transport limitations, stromal constituents, particularly CAFs, actively orchestrate therapy resistance through paracrine signaling and direct cell–cell contacts. CAFs secrete a broad repertoire of growth factors, cytokines, and ECM-remodeling enzymes that create a drug-resistant niche (135, 136). For instance, CAF-derived hepatocyte growth factor (HGF) can reactivate alternative survival pathways in tumor cells and has been implicated in resistance to targeted therapies; in BRAF V600E mutant melanomas, stromal fibroblast secretion of HGF activates MET signaling in melanoma cells, engaging MAPK/PI3K pathways that blunt BRAF inhibitor efficacy. CAF-secreted IL-6 and IL-8 can similarly activate STAT3 and other pro-survival programs, conferring broader chemoresistance and promoting stem-like traits (137). Moreover, adhesion of tumor cells to ECM components such as fibronectin or VCAM-1 often termed cell-adhesion-mediated drug resistance, delivers anti-apoptotic survival signals that help tumor cells withstand chemotherapy (138). Importantly, this protective niche effect can be dynamic and reversible: tumor cells removed from stromal influence may regain drug sensitivity, underscoring that microenvironmental support is an ongoing requirement for tumor persistence under therapeutic pressure. Collectively, CAF-driven paracrine and adhesion-dependent programs position stromal cells as “co-conspirators” that actively shield malignant cells from therapy-induced stress (135).

### 5.3 Adaptive resistance and therapy-Induced TME remodeling

Therapeutic pressure can dynamically reprogram stromal, vascular, and immune compartments, generating adaptive resistance through therapy-induced TME remodeling. Importantly, the influence of the TME on therapy response is bidirectional: therapies themselves can reshape the microenvironment in ways that promote tumor survival, a phenomenon observed across treatment modalities (139). For instance, chemo- and radiotherapy can trigger wound-healing programs in residual disease, activating fibroblasts and myeloid cells and creating a pro-repair niche that supports tumor persistence. Intravital imaging in breast cancer models showed that cytotoxic chemotherapy enriched stromal cells around drug-tolerant tumor clusters, with macrophages accumulating and releasing mediators (including IL-1β and cathepsins) that promoted regrowth and blunted subsequent chemotherapy effects (140). After radiotherapy, surviving regions frequently exhibit increased fibrosis (driven by TGF-β from irradiated stromal cells) together with recruitment of M2-polarized macrophages, forming a milieu conducive to regrowth and radioresistance (141). Anti-angiogenic therapy provides another example: while VEGF blockade may transiently normalize vessels, adaptive vascular remodeling can ultimately intensify hypoxia and redirect tumor evolution toward invasive phenotypes. In glioblastoma, VEGF inhibition was associated with invasive tumor fronts accompanied by accumulation of Tie2-expressing monocytes, indicating microenvironment-driven adaptation rather than durable control (142).

Adaptive TME responses are also pivotal during targeted therapies and immunotherapy. Targeted kinase inhibition may initially shrink tumors, yet stromal feedback loops can restore survival signaling. For example, in BRAF-mutant melanoma, BRAF inhibitors can paradoxically activate RAF/ERK signaling in adjacent fibroblasts, inducing secretion of growth factors (e.g., FGF, EGF, NRG1) that reactivate bypass pathways in melanoma cells and confer resistance (143). With ICIs blockade, responding tumors may develop acquired resistance through microenvironmental reprogramming, including reinforcement of inhibitory metabolites and checkpoints (e.g., PD-L1, IDO, adenosine) and expansion of stromal programs that exclude or suppress T cells. Consistent with this, non-responding melanoma biopsies have been reported to show a “cold” milieu enriched in activated fibroblasts and TGF-β signaling compared with responders, implicating a TGF-β–dominated stroma in immune escape (143). Similarly, in urothelial carcinoma, a CAF/TGF-β–rich signature correlates with primary resistance to PD-1/PD-L1 blockade, supporting the concept that activated stroma can nullify otherwise potent immunotherapy (144). These observations provide a rationale for combination strategies that pair ICIs with agents that block TGF-β signaling or reprogram CAFs to prevent or overcome therapy-induced microenvironmental resistance.

### 5.4 Representative tumor contexts illustrating TME-mediated resistance

Certain malignancies are paradigms of TME-driven therapeutic failure. Pancreatic ductal adenocarcinoma is typically surrounded by a dense fibrotic stroma that elevates interstitial pressure and severely limits drug perfusion (145). This is one reason gemcitabine has historically shown poor penetration and efficacy in pancreatic tumors (146). In addition, pancreatic TAMs can deactivate gemcitabine, and fibroblasts expressing fibroblast activation protein-α (FAP) form an immunosuppressive mesh that restricts T-cell infiltration (147). Preclinical studies further demonstrated that depleting FAP+ stromal cells or enzymatically dismantling stromal barriers can improve drug delivery and enable immune attack in pancreatic cancer (148). Triple-negative breast cancer (TNBC) provides another instructive example: although TNBC can exhibit substantial immune infiltration, the infiltrate is often enriched for pro-tumorigenic macrophages and neutrophils. TAMs have been implicated in blunting chemotherapeutic responses (e.g., via IL-10 and cathepsins) and in supporting stem-like tumor cell programs linked to relapse (140). Clinically, high macrophage content predicts poorer response to neoadjuvant chemotherapy, whereas approaches that reprogram macrophages (e.g., CD40 agonism) or limit their recruitment can improve chemotherapy efficacy (149). Melanoma, while often immunogenic and responsive to checkpoint blockade, still exhibits TME-mediated resistance: inflammatory cytokines (e.g., TNF-α, IL-8) can induce a reversible drug-tolerant state, and CAF-derived factors (including HGF and TGF-β) contribute to both targeted-therapy resistance and immune evasion, as discussed above (143). Importantly, TME-driven resistance is not limited to these “classic” examples; even in malignancies historically considered relatively chemo-sensitive (e.g., ovarian cancer) or therapeutically heterogeneous (e.g., soft tissue sarcomas), relapse after initial response is frequently attributed to microenvironmental protection, such as peritoneal “niches” that shield ovarian cancer cells or MSC/M2-macrophage-rich contexts that support sarcoma regrowth.

Beyond desmoplastic tumors, additional cancer types further illustrate how distinct TME programs constrain therapy. Colorectal cancer highlights a key dichotomy: while dMMR/MSI-H tumors are typically more immunogenic and often derive marked benefit from PD-1 blockade (150), the more common pMMR/MSS subtype frequently exhibits immune exclusion driven by stromal/TGF-β programs and myeloid suppression, limiting durable responses to checkpoint therapy (151, 152). Prostate cancer commonly shows low baseline T-cell infiltration with dominant suppressive myeloid/stromal signaling, contributing to relative resistance to ICI monotherapy (153). Hepatocellular carcinoma is shaped by a tolerogenic hepatic milieu and can feature hypoxia, aberrant vasculature, and suppressive macrophage networks that jointly blunt immune-mediated control (154). Across these diverse tumor types, a recurring theme is that shared TME features, fibrosis, abnormal vessels, hypoxia/acidosis, and suppressive immune circuits, can underlie resistance to otherwise distinct treatment modalities.

Tumor antigenicity, often approximated by TMB and neoantigen burden, is a major determinant of responsiveness to immune checkpoint inhibition. Across clinical cohorts treated with ICIs, higher TMB has been associated with improved outcomes in multiple cancer types (155, 156), and high—particularly clonal—neoantigen burden correlates with stronger T-cell immunoreactivity and greater sensitivity to checkpoint blockade (157). However, antigenicity alone is not sufficient: in immune-excluded tumors, stromal and vascular constraints (especially TGF-β–dominated programs) can physically sequester T cells in peritumoral stroma and prevent access to tumor nests, effectively neutralizing neoantigen-specific immunity despite the presence of targets (151, 152). Thus, durable benefit from ICIs most often requires alignment of both antigenicity (TMB/neoantigens) and immune accessibility (infiltration/positioning and functional competence), providing a rationale for combination strategies that pair ICIs with stromal/TGF-β-targeting interventions in antigenic but immune-excluded tumors.

## 6 Therapeutic strategies to reprogram the tumor microenvironment

Tumors co-opt their microenvironment to evade therapy, but this dependency offers therapeutic opportunities. Targeting the non-malignant stromal components of the TME, such as fibroblasts, vasculature, immune cells, and metabolic factors, can reprogram the TME to be more permissive to therapy and immunosurveillance. Below are condensed summaries of therapeutic strategies that aim to overcome resistance by modifying the TME. Importantly, tumor-associated inflammation is not uniformly tumor-promoting. Chronic, dysregulated inflammatory signaling can fuel fibrosis and immune suppression (e.g., via IL-1β/IL-6/TNF-driven myeloid recruitment, M2-like TAM polarization, and fibro-inflammatory crosstalk that amplifies ECM deposition), thereby reinforcing stromal barriers and therapy resistance (158). Conversely, appropriately oriented inflammatory programs can be tumor-suppressive by promoting antigen presentation, type I/II interferon signaling, and recruitment/activation of cytotoxic CD8+ T cells and NK cells. Therefore, anti-fibrotic interventions should ideally re-balance inflammatory circuits, attenuating pathological fibro-inflammatory loops while preserving (or restoring) productive, antitumor inflammation, rather than broadly extinguishing inflammation (159).

While multiple therapeutic strategies aim to reprogram the tumor microenvironment, their clinical translation remains uneven. Major biological and translational limitations across TME-targeting approaches are summarized in **Table 3**.

**Table 3:** Key controversies and translational gaps in tumor microenvironment reprogramming.

Domain	What is well-supported	Key controversy/limitation	Practical implication
CAF targeting	ECM/TGF-β barrier in excluded tumors	CAF depletion can be harmful; subtype heterogeneity	Prefer reprogramming, subtype biomarkers
Myeloid targeting	TAM/MDSC suppress T cells	marker heterogeneity; mixed trial results	Harmonize definitions; combos + timing
Metabolic targeting	Adenosine/hypoxia suppress function	limited clinical translation	patient selection via signatures
Anti-angiogenic combos	can normalize vessels	“normalization window” and toxicity	dosing/schedule critical
Checkpoint beyond PD-1	LAG-3/TIM-3 etc.	context-dependent benefit	avoid one-size-fits-all

### 6.1 Anti-fibrotic approaches

Desmoplasia driven by CAFs creates a physical and biochemical barrier to treatment (160). Inflammation-driven CAF states underscore the context-dependent, dual role of tumor-associated inflammation in fibrosis and therapy response. In rectal cancer, Nicolas and colleagues reported that inflammatory CAFs (iCAFs) are associated with poor response to neoadjuvant chemoradiotherapy and that IL-1α signaling promotes a therapy-induced, senescence-linked iCAF program with ECM accumulation that supports resistance (161). Consistently, IL-1/IL-1R pathway blockade (e.g., IL-1 receptor antagonism) attenuated this pro-tumorigenic fibro-inflammatory state and shifted CAF programs toward a more tumor-restraining phenotype, providing a rationale for combining anti-fibrotic strategies with pathway-selective anti-inflammatory intervention in desmoplastic tumors (162). Rather than indiscriminate CAF ablation, reprogramming approaches using, for example, TGF-β pathway inhibitors or vitamin analogs, have been proposed to modulate stromal barriers while preserving potentially tumor-restraining fibroblast functions (123). In murine models, TGF-β blockade reduces fibrotic constraints, restores intratumoral T-cell infiltration, and can synergize with checkpoint inhibition (163). Similarly, targeting the CXCL12–CXCR4 axis (e.g., plerixafor) has increased cytotoxic T-cell access and enhanced immunotherapy efficacy in preclinical settings (164).

A central translational challenge is the marked heterogeneity and plasticity of CAFs, which comprise functionally distinct states (e.g., inflammatory, myofibroblastic, antigen-presenting programs) that can interconvert under therapeutic and inflammatory cues (159). Accordingly, broad anti-fibrotic strategies or nonspecific CAF depletion may produce variable effects and, in some contexts, inadvertently remove fibroblast subsets that restrain tumor growth or facilitate immune cell entry. These considerations support state-selective CAF modulation (e.g., targeting IL-1–driven iCAF circuits or TGF-β–linked myofibroblastic programs) coupled with biomarker-guided patient stratification, rather than uniform CAF ablation, to maximize therapeutic benefit and minimize unintended consequences (165).

CAFs, TAMs, MDSCs, and Tregs are often discussed as uniformly tumor-promoting; however, accumulating evidence supports marked heterogeneity and context dependence that limits one-size-fits-all targeting. CAFs, in particular, encompass multiple functional states (e.g., inflammatory versus myofibroblastic programs) that can interconvert under inflammatory and therapeutic cues, and certain fibroblast subsets may restrain tumor progression or facilitate immune access in specific settings (159). Accordingly, broad stromal depletion has yielded paradoxical outcomes in some preclinical models, including accelerated disease and reduced survival, underscoring that stromal compartments can function both as barriers to immunity and, in defined contexts, as constraints on tumor evolution depending on tumor type, stage, and treatment setting (166). Similar context dependence extends to myeloid and regulatory populations: macrophage polarization and suppressive networks are dynamic, and interventions may produce divergent effects based on timing, baseline immune phenotype, and dominant cytokine or metabolic programs (167). Together, these observations motivate state-selective TME reprogramming and highlight priority gaps, including (i) establishing causal CAF lineages/states in patients, (ii) developing biomarkers that distinguish tumor-restraining versus tumor-promoting stromal programs, and (iii) mapping temporal remodeling under therapy to inform rational combination design.

Despite strong mechanistic rationale, stromal/ECM-directed approaches have shown heterogeneous clinical translation, emphasizing the need for biomarker-guided selection and careful scheduling. For example, enzymatic hyaluronan depletion with pegylated hyaluronidase (PEGPH20) in hyaluronan-high metastatic pancreatic cancer did not improve overall survival when added to gemcitabine/nab-paclitaxel in a phase III trial, illustrating that stromal remodeling does not uniformly convert into durable clinical benefit (168). Moreover, TGF-β signaling is context-dependent and can be both tumor-promoting and tumor-suppressive depending on tumor type and stage; therefore, indiscriminate or excessive pathway inhibition may yield divergent outcomes across models, including reports of enhanced invasive/metastatic behavior in some settings (169). These complexities support state-selective stromal reprogramming and biomarker-guided combinations rather than uniform stromal depletion.

Representative clinical studies evaluating anti-fibrotic and CAF-/stroma-directed strategies to reprogram the tumor microenvironment are summarized in **Supplementary Table 3** (trial identifiers provided where available).

### 6.2 Vascular normalization strategies

Tumor vasculature is often disorganized and hypoxic, hindering drug delivery and immune infiltration (170). Anti-angiogenic therapy (e.g., bevacizumab) can transiently normalize vessels, improving perfusion and potentiating chemotherapy or immunotherapy (171). Clinical benefits of such monotherapies are modest, but combinations, particularly VEGF inhibitors with ICIs, have yielded improved outcomes in cancers like HCC and RCC (172–174). Optimal dosing and biomarker-driven scheduling remain active areas of research (12). However, it is important to note that VEGF/VEGFR2 blockade has shown heterogeneous and often transient benefits: in orthotopic mouse models, anti-angiogenic therapy did not consistently achieve durable tumor control and, in some settings, was associated with adaptive resistance and invasive phenotypes (175). Moreover, in clinical studies, VEGF or VEGFR2 inhibitors have been clearly effective only in a subset of tumor types and typically yield limited-duration benefit rather than broadly durable responses (176, 177).

### 6.3 Myeloid cell reprogramming

TAMs and MDSCs suppress anti-tumor immunity (178). CSF-1R inhibitors can deplete TAMs or reprogram them toward a pro-inflammatory phenotype, enhancing chemotherapy and ICI responses (179). CD40 agonists also activate macrophages and DCs to improve T-cell infiltration (180). Although monotherapies show limited efficacy, combinations with ICIs are under clinical investigation, particularly in immunologically cold tumors like pancreatic cancer (181, 182).

### 6.4 Targeting metabolism and pH

Metabolic competition and acidosis within the TME impose major constraints on antitumor immunity, limiting effector function and reinforcing immunosuppression (183). Accordingly, metabolic reprogramming interventions are increasingly explored to restore T-cell activity in metabolically suppressive TMEs (49). One major axis is inhibition of the IDO–kynurenine pathway: although IDO inhibitors were initially promising, clinical setbacks have prompted trials with more potent agents and rational combinations (78, 184). In parallel, adenosine pathway blockade (e.g., A2A receptor antagonists and anti-CD73 antibodies) aims to reverse adenosine-mediated suppression and restore T-cell function in tumors with high extracellular adenosine (50, 91, 185). Additional strategies target arginine depletion driven by suppressive myeloid programs, including arginase inhibition to increase intratumoral arginine availability and improve T-cell proliferation and function (79). Approaches to modulate tumor acidity, such as buffering strategies (e.g., bicarbonate) or inhibition of pH-regulating enzymes like carbonic anhydrase IX (CAIX)—have shown preclinical efficacy and are advancing into clinical evaluation (91, 185). Finally, efforts to enhance T-cell metabolic fitness more directly (e.g., dietary or cytokine-based support) are under investigation, although much of this remains early-stage (80). Collectively, these metabolic and pH-directed interventions are frequently being tested in combination with immune checkpoint inhibitors, with the goal of strengthening functional antitumor immunity and potentially converting non-responders into responders (186).

### 6.5 Combination therapies with ICIs

Encouragingly, T cell exhaustion is not absolute and can be therapeutically reversed to a degree. The clinical success of checkpoint inhibitors such as anti-PD-1 demonstrates that even dysfunctional T cells can be “reinvigorated”; importantly, PD-1 blockade can restore proliferative capacity in a subset of exhausted T cells, particularly less-differentiated progenitor-like Tex, and partially recover tumor-killing functions (53). Consistent with this, tumors with a baseline T cell-inflamed phenotype tend to respond better to checkpoint therapy, suggesting that Tex retain actionable antitumor potential when appropriately unleashed.

These observations have motivated combinatorial checkpoint approaches aimed at releasing multiple inhibitory constraints simultaneously. CTLA-4 inhibition (e.g., ipilimumab) can enhance priming and mitigate intratumoral regulatory circuits, while PD-1 inhibition (e.g., nivolumab) reinvigorates pre-existing exhausted T cells; their combination has improved outcomes in melanoma and other cancers (48). More recently, LAG-3 blockade combined with PD-1 inhibition (e.g., relatlimab plus nivolumab) has also shown clinical benefit in melanoma (48), and additional dual or triple combinations (including PD-1 with TIGIT or TIM-3 blockade) remain under active investigation.

Because ICIs are most effective in TMEs that permit immune infiltration and activation, they are increasingly paired with TME-directed interventions to overcome resistance (154, 187). Representative strategies include TGF-β or CXCR4 inhibition to improve T-cell access, anti-angiogenic approaches to alleviate hypoxia-associated suppression, and myeloid-targeting agents to relieve dominant inhibitory circuits. Across these combinations, biomarker-guided patient selection and careful management of additive toxicity are critical for maximizing benefit while limiting harm (188, 189).

Despite these advances, several challenges limit the impact of ICIs in many solid tumors, particularly when the baseline TME is TGF-β–dominated and immune-excluded (12). In such contexts, checkpoint blockade may fail because T cells are physically and functionally restrained by stromal and vascular barriers and by TGF-β–driven suppressive programs, leaving insufficient tumor-reactive cells to be effectively reinvigorated. Moreover, across cancer types, only a minority of patients achieve durable clinical benefit from ICIs, underscoring the need for biomarker-guided patient selection and rational combination strategies that may, in selected settings, help shift ‘cold’ or immune-excluded tumors toward more inflamed phenotypes (190). Finally, therapeutic T-cell activation can trigger immune-related adverse events (irAEs), ranging from mild inflammatory toxicities to severe, autoimmune-like manifestations that may require immunosuppression and can limit dose intensity or treatment continuation.

### 6.6 Emerging approaches: oncolytic viruses, nanoparticles, and bispecific antibodies

Beyond established strategies, a new generation of therapeutic platforms seeks to modify the TME with greater specificity, multifunctionality, and spatial precision. These modalities include oncolytic viruses (OVs), nanotechnology-based delivery systems, and bispecific antibodies (BsAbs). Oncolytic viruses (e.g., T-VEC) induce local inflammation and can enhance systemic antitumor immunity (191). Nanoparticles enable localized delivery of immunomodulators or stromal-targeting drugs, improving safety and efficacy (192). Innate immune agonists represent an additional strategy to convert “cold” or immune-excluded TMEs toward productive inflammation. STING and TLR agonists can promote type I interferon programs, enhance dendritic-cell activation and antigen presentation, and increase chemokine-driven recruitment of effector lymphocytes mechanistically complementing checkpoint blockade (193). Clinically, these agents have most commonly been explored as intratumoral or localized approaches and frequently in combination with ICIs, although activity remains context-dependent and limited by delivery and inflammatory toxicities, highlighting the need for careful scheduling and spatially guided administration (194). Bispecific antibodies can redirect immune cells toward tumor and stromal antigens or co-block immune checkpoints, and several agents are under clinical evaluation (195, 196). Collectively, these platforms enable multifunctional and spatially precise TME modulation and expand the toolkit for phenotype-matched combination strategies.

#### 6.6.1 Oncolytic viruses

OVs are engineered or naturally occurring viruses that selectively infect and lyse cancer cells, releasing tumor antigens and stimulating local immune activation. Through oncolysis and local immunogenic cell death, oncolytic viruses can enhance antigen release and presentation and promote local type I interferon–driven inflammation. In selected contexts, these effects may increase intratumoral immune infiltration and improve responsiveness to immunotherapies, particularly when combined with ICIs.Talimogene laherparepvec (T-VEC), an HSV-1–based OV encoding GM-CSF, was the first FDA-approved oncolytic agent, showing improved durable response rates in advanced melanoma (197). OVs promote DCs activation and T-cell priming, with abscopal effects observed in some trials. Combinations of OVs with checkpoint inhibitors are being tested in melanoma, triple-negative breast cancer, and glioblastoma (198). However, antiviral immunity and tumor delivery pose challenges. To address these, strategies such as repeated intratumoral dosing, nanoparticle encapsulation, and cell-mediated delivery (e.g., mesenchymal stem cells loaded with virus) are under investigation (199).

#### 6.6.2 Nanoparticle-based delivery systems

Nanoparticles (NPs) offer a platform for delivering immunomodulatory or cytotoxic payloads directly to the TME, exploiting enhanced permeability and retention, as well as TME-specific cues like pH, enzymatic activity, or redox potential. Multifunctional NPs have been designed to co-deliver chemotherapy and stromal-targeting agents (e.g., TGF-β inhibitors or siRNAs), simultaneously weakening stromal barriers and killing tumor cells (200). Some NPs promote vascular normalization or reprogram tumor-associated macrophages toward an M1 phenotype. Tumor-activated nanocarriers release cargo in response to environmental triggers, minimizing off-target toxicity. Several FDA-approved nanomedicines (e.g., liposomal doxorubicin, albumin-bound paclitaxel) demonstrate the clinical utility of this approach, while newer formulations are entering trials (201, 202).

#### 6.6.3 Bispecific antibodies

BsAbs are engineered antibodies capable of simultaneously binding two targets. T-cell engagers (e.g., CD3×tumor antigen) recruit and activate T cells at the tumor site, bypassing the need for MHC-dependent antigen presentation. Blinatumomab (CD19×CD3) is approved in hematologic malignancies, and analogous agents targeting solid tumor antigens (e.g., PSMA, EGFR) are in clinical development (203). BsAbs also include dual checkpoint inhibitors (e.g., PD-1×CTLA-4), tumor-targeted cytokine fusions (e.g., IL-2 or IL-12 localized via FAP-binding domains), and CD137 agonists activated only in the tumor stroma. These designs enhance immune activation while minimizing systemic toxicity. One innovative format fuses immune costimulatory agonists with ECM-targeting arms to focus activation within the TME (72). Challenges include large size, tumor penetration, and cytokine release, which are being addressed through step-dosing, conditional activation, and structural engineering.

In summary, these emerging platforms expand the repertoire of TME-directed therapies. Their precision, modularity, and combinatorial potential make them promising tools to overcome therapy resistance and improve immunotherapy responsiveness.

## 7 Discussion and future perspectives

The literature consistently shows that diverse TME components cooperate to blunt anti-cancer therapies. Dense fibrotic stroma physically impedes drug and immune cell penetration: for example, desmoplastic collagenous matrices form a protective “cocoon” around tumor cells, shielding them from cytotoxic agents and host immunity (204). Abnormal tumor vasculature further exacerbates resistance – tortuous, leaky vessels induce hypoxia and high interstitial fluid pressure, which not only reduce drug delivery but also promote radioresistance and metastatic signaling (205, 206). Immunosuppressive stromal and immune cells supply another layer of defense. TAMs, MDSCs and regulatory T cells secrete TGF-β, IL-10 and other factors that inactivate cytotoxic T cells and natural killer cells (207, 208). Metabolic reprogramming in the TME compounds these effects: hypoxia and glycolysis-driven acidosis impair effector T cell function, and high lactate levels promote M2-like TAM polarization and DCs dysfunction (207, 208). Collectively, these TME features, fibrosis, aberrant vessels, suppressive immune cells and hostile metabolism, establish a multifaceted barrier, broadly driving resistance to chemotherapy, targeted therapy, radiotherapy and immunotherapy (209).

Importantly, the same TME that protects tumors also offers therapeutic targets. Anti-angiogenic drugs (e.g. VEGF inhibitors) have entered the clinic precisely to exploit abnormal vasculature; judicious use can “normalize” vessels transiently, improving perfusion and the efficacy of chemo- and immunotherapies (205). ICIs (targeting PD-1/PD-L1, CTLA-4, etc.) act in part by shifting the TME from suppressive to immunostimulatory. Emerging strategies directly disrupt stromal support: for instance, TGF-β blockade or CXCL12/CXCR4 inhibitors can reprogram fibroblasts and dissolve immune-excluding matrices, and CSF1R antagonists attempt to deplete protumor TAMs. Preclinical evidence suggests that combining conventional drugs with TME modulators – for example, adding CXCR4 or TGF-β inhibitors to chemotherapy and checkpoint blockade can overcome microenvironmental resistance (210, 211). Likewise, novel immune-engineered therapies (such as CAR-T cells or bispecific antibodies directed against stromal antigens like FAP) are being developed to penetrate the TME; early studies of CAF-targeted CAR-T and stromal vaccines show promising activity in models (212). Indeed, tumor stromal elements are being successfully imaged and targeted in patients (e.g. FAP-directed tracers), underscoring the clinical relevance of the TME as a therapeutic focus (213). In short, the TME is not merely an inert barrier but a dynamic ecosystem that can be manipulated for therapy (echoing Virchow and Balkwill’s notion that tumor-associated inflammation fuels cancer (214), now understood as an actionable vulnerability).

Despite these advances, translational challenges remain. Chief among them is the striking heterogeneity of stromal cells. CAFs exist in multiple subtypes (myofibroblastic, inflammatory, antigen-presenting, etc.), each with distinct functions; pan-depletion has proven risky since some CAF subsets restrain rather than promote tumor growth (215). Similarly, TAMs and other myeloid cells form a continuum of activation states beyond the simple M1/M2 paradigm. Their plasticity and lack of unique markers complicate efforts at selective targeting (216). In practice, this means that indiscriminate targeting can have unintended effects (for example, ablation of FAP+ CAFs actually accelerated pancreatic tumor growth in mice (215), and that robust biomarkers are needed to identify which patients harbor a given TME vulnerability. Currently, few reliable biomarkers exist to stratify patients for TME-directed therapies: efforts at defining stromal signatures (e.g. collagen gene sets, immune-exclusion gene profiles) or imaging TME features (e.g. FAPI PET) are ongoing but not yet validated in trials. Physical delivery also poses a hurdle: dense ECM and high pressure impede diffusion of large molecules or cells, necessitating specialized drug carriers or co-treatments (enzymes, vascular modulators) (215). Finally, off-target toxicity remains a concern, since many TME antigens (e.g. FAP, CXCR4) are shared with normal tissues. In sum, the complex, patient-specific nature of the TME, and the current lack of precise biomarkers and delivery techniques, limits translation of stromal targets into broadly effective treatments (216).

An additional, host-level layer shaping antitumor immunity is the human microbiome. Growing evidence indicates that gut microbial composition and metabolites can modulate systemic and intratumoral immune tone by influencing DCs maturation, antigen presentation, and effector T-cell priming, thereby affecting the efficacy and potentially toxicity of ICIs (217). Clinically, microbiome features have been associated with differential responses to PD-1/PD-L1 therapy across tumor types, and interventional approaches such as fecal microbiota transplantation (FMT) have demonstrated that microbiome modulation can overcome anti–PD-1 resistance in subsets of melanoma patients, supporting a causal role in at least some settings (218, 219). These observations motivate future biomarker-guided strategies that integrate microbiome profiling with TME phenotyping to refine patient selection and rationally combine ICB with microbiome-directed interventions.

Therapy resistance remains a central challenge and should be viewed as an emergent, dynamic process spanning tumor-intrinsic alterations, TME-extrinsic suppression, and host determinants. Tumor-intrinsic mechanisms include impaired antigen presentation (e.g., loss of HLA/B2M), defects in interferon signaling, and activation of survival pathways that blunt immune-mediated killing. In parallel, TME-driven resistance arises from impaired T-cell trafficking (stromal/vascular exclusion), dominant immunosuppressive circuits (TAM/MDSC/Treg networks, TGF-β), and metabolic constraints (hypoxia, adenosine, nutrient competition) that enforce dysfunctional effector states (220). Importantly, resistance can be primary (innate) or acquired, with therapeutic pressure actively remodeling the microenvironment and selecting for resistant tumor cell states. Collectively, these considerations support phenotype-matched combination regimens and adaptive sequencing approaches that target the dominant resistance axis, access/trafficking barriers in EXC versus functional/metabolic/inhibitory barriers in immune-inflamed but dysfunctional or exhausted states (INF-D/EXH), rather than applying uniform strategies across biologically distinct TMEs (221).

Future directions to overcome these barriers are multifaceted:

- Spatial and single-cell multi-omics: High-resolution mapping of the TME will be crucial. Cutting-edge platforms (spatial transcriptomics, multiplex imaging, single-cell RNA-seq) can delineate stromal and immune subsets *in situ* (222, 223). Comprehensive “cell atlases” of CAFs, TAMs, T cells and endothelial cells within tumors will allow identification of the dominant resistance programs in each cancer. This deep profiling can also reveal novel intercellular circuits (e.g. metabolite exchange, ligand–receptor pairs) to target. For example, integrating single-cell genomics with spatial imaging has already uncovered distinct CAF niches and their associations with therapy outcomes (224).
- Personalized TME interventions: Tumors should be classified by their TME phenotype to guide therapy. Immune “hot” (inflamed) cancers may primarily need checkpoint blockade, whereas “cold” tumors with dense fibrosis or exclusion (e.g. pancreatic, some breast/ovarian carcinomas) may require stromal-modifying first. Converting immune-deserts into inflamed phenotypes is a priority (225). This could involve patient-tailored combinations, for instance, using collagenase or hyaluronidase in fibrotic tumors to open up T cell access, or deploying bispecifics that simultaneously engage tumor and stromal antigens only in defined contexts. The concept of matching TME-modifying drugs to the tumor’s ecosystem (fibrotic vs. immune-excluded vs. inflamed) represents a promising precision medicine approach.
- Novel combination modalities: TME-targeting should be integrated with emerging therapies. CAR-T and T cell-engaging bispecifics are being adapted to attack stromal cells (for example, anti-FAP CAR-T or dual-antigen CARs against tumor+fibroblast markers) (226). Likewise, oncolytic viruses or “armored” T cells that secrete TME-modulating cytokines could reshape the microenvironment. An exciting frontier is microbiome modulation: gut and intratumoral microbes can profoundly influence the TME and therapy response, so interventions like FMT, probiotics, or diet may become adjuvants to immunotherapy (227). Finally, theranostic approaches using advanced imaging or biomaterials will help monitor TME changes in real time and deliver payloads selectively.
- Emerging enabling technologies and innate immune modulation: Recent advances in spatially resolved profiling—particularly spatial transcriptomics and multiplex imaging are reshaping how immune landscapes are defined in solid tumors. By preserving tissue architecture while quantifying cell states and interaction networks, these technologies can distinguish true immune exclusion from mere low cellularity, resolve compartmentalized phenotypes (e.g., T cells trapped in stroma vs infiltrating tumor nests), and map functional niches such as hypoxic/adenosine-rich zones (228). Importantly, spatial approaches enable identification of clinically relevant cellular neighborhoods (e.g., CAF–myeloid–endothelial “barrier” niches) that may not be apparent in bulk profiling and can guide biomarker-driven therapy selection and rational combination design (229, 230).

In conclusion, therapeutically “taming” the TME represents a critical next step in oncology. With the advent of sophisticated omics tools and modular immunotherapies, we anticipate a shift from viewing the stroma as an untouchable fortress to treating it as a tractable partner. Strategically directed combinations – for example, checkpoint blockade plus fibroblast reprogramming, or targeted vasculature normalization with adoptive T cells – are poised to yield deeper, more durable remissions. By integrating TME profiling into clinical decision-making and by innovating multi-modal strategies, future therapies will increasingly exploit the microenvironment. Ultimately, unlocking the TME’s therapeutic potential may allow us to convert resistant tumors into responsive ones, achieving broader and more lasting responses in cancer patients.

## 8 Conclusion

Tumor immune landscapes are increasingly recognized as dynamic, evolving systems that critically shape therapeutic responsiveness. One major trajectory is the shift from immune exclusion to immune exhaustion, which reflects how tumor–host interactions drive both primary resistance and acquired treatment failure. Importantly, these immune states are not strictly discrete. Immune-excluded and immune-exhausted features can coexist within the same tumor, and they may also arise sequentially under therapeutic pressure, highlighting the plasticity of the tumor microenvironment.

This dynamic view has direct clinical implications. Therapeutic failure is often not explained by tumor-intrinsic resistance alone. Instead, adaptive remodeling of stromal, vascular, and immune compartments can dampen effective antitumor immunity and limit durable benefit. Accordingly, the success of immunotherapy and combination regimens depends not only on tumor mutational features, but also on the ability to overcome exclusion, reverse T-cell dysfunction, and attenuate suppressive microenvironmental programs.

Future strategies should therefore move beyond one-size-fits-all immunomodulation and adopt context-adaptive interventions. These approaches should be guided by spatial, functional, and temporal profiling of the tumor microenvironment. Integrating immune phenotyping with stromal architecture and metabolic constraints may enable rational sequencing and combination of immunotherapies, anti-angiogenic agents, and microenvironment-targeted treatments. Ultimately, reframing cancer therapy through the lens of TME dynamics may convert immune resistance from a barrier into a therapeutic opportunity.
