# Supplementary material for: From immune exclusion to exhaustion: tumor microenvironment drives therapy response
Source: Front Immunol. 2026 Apr 22;17:1785587. doi: 10.3389/fimmu.2026.1785587 (PMC13144129; doi:10.3389/fimmu.2026.1785587)
Supplement: Supplementary file 1 [file Table1.docx]

| Strategy / axis | Agent(s) | Tumor type / setting | Phase | Trial ID |
| --- | --- | --- | --- | --- |
| TGF-β pathway inhibition + PD-1 blockade | Vactosertib (TGF-βR1 inhibitor) + pembrolizumab | Metastatic microsatellite-stable colorectal cancer (MSS mCRC) / gastric cancer | Phase 1b/2a | NCT03724851 |
| TGF-β pathway inhibition + PD-1 blockade | Vactosertib + pembrolizumab | 1st-line advanced/metastatic PD-L1+ NSCLC | Phase 2 | NCT04515979 |
| Adenosine A2A receptor blockade ± PD-L1 blockade | Ciforadenant (A2AR antagonist) ± atezolizumab | Advanced solid tumors (registry-defined cohorts) | Phase 1/1b | NCT02655822 |
| CD73 blockade (adenosine axis) + PD-L1 blockade (consolidation) | Oleclumab (anti-CD73) + durvalumab | Unresectable stage III NSCLC, post-cCRT consolidation (platform) | Phase 2 | NCT03822351 |
| CD73 blockade (adenosine axis) + PD-L1 blockade (consolidation) | Oleclumab + durvalumab | Unresectable stage III NSCLC, post-cCRT consolidation | Phase 3 | NCT05221840 |
| Chemokine axis / trafficking (CXCR4) in combination regimens | Motixafortide (CXCR4 antagonist) + cemiplimab + gemcitabine/nab-paclitaxel | Metastatic pancreatic ductal adenocarcinoma (mPDAC) | Phase 2 | NCT04543071 |

**Supplementary Table S1.** Selected clinical trials targeting immune exclusion and immunosuppressive axes (trial identifiers provided)

**Supplementary Table S2.** Selected clinical trials “anchor” studies for angiogenesis-hypoxia-metabolic targeting strategies (trial identifiers provided)

| Therapeutic domain | Regimen | Tumor type / setting | Phase | Trial ID |
| --- | --- | --- | --- | --- |
| Anti-angiogenic + ICI (vascular normalization–informed combinations) | Atezolizumab + bevacizumab vs sorafenib (IMbrave150) | Unresectable / advanced hepatocellular carcinoma (uHCC) | Phase 3 | NCT03434379 |
| Anti-angiogenic + ICI | Pembrolizumab + axitinib vs sunitinib (KEYNOTE-426) | 1st-line advanced renal cell carcinoma (RCC) | Phase 3 | NCT02853331 |
| Anti-angiogenic + ICI | Nivolumab + cabozantinib vs sunitinib (CheckMate 9ER) | 1st-line advanced RCC | Phase 3 | NCT03141177 |
| Anti-angiogenic + ICI | Lenvatinib + pembrolizumab (Study 116 / KEYNOTE-524) | Unresectable HCC (uHCC), early-phase safety/efficacy | Phase 1b | NCT03006926 |
| Hypoxia-targeted therapy (hypoxia-activated prodrug) + standard therapy | Evofosfamide (TH-302) + preoperative chemoradiotherapy | Esophageal cancer (preoperative chemoradiotherapy setting) | Phase 1 | NCT02598687 |
| Metabolic targeting (glutamine axis) + standard ICI/chemo | Telaglenastat (glutaminase inhibitor) + pembrolizumab + chemotherapy (KEAPSAKE) | 1st-line non-squamous NSCLC with KEAP1/NRF2 alterations (registry-defined) | Phase 2 | NCT04265534 |

**Supplementary Table S3**. Selected clinical studies of anti-fibrotic/CAF- and stroma-targeting strategies to reprogram the tumor microenvironment

| Strategy / target (anti-fibrotic / CAF / stroma) | Agent(s) / regimen | Tumor type / setting | Phase | Trial ID |
| --- | --- | --- | --- | --- |
| IL-1R blockade to modulate fibro-inflammation (CAF state reprogramming concept aligned with iCAF biology) | Anakinra (IL-1 receptor antagonist) + capecitabine-based chemoradiotherapy | Locally advanced rectal cancer; neoadjuvant CRT | Phase I | NCT04942626 (also registered as DRKS00025477) ([ClinicalTrials.gov](https://clinicaltrials.gov/study/NCT04942626?utm_source=chatgpt.com)) |
| FAP-targeted immunocytokine (CAF/stroma-directed immune activation) | RO6874281 / simlukafusp alfa (FAP-IL2v), monotherapy or combinations (platform) | Advanced/metastatic solid tumors (accessible lesions) | Phase I | NCT02627274 ([ClinicalTrials.gov](https://clinicaltrials.gov/study/NCT02627274?utm_source=chatgpt.com)) |
| FAP-targeted immunocytokine + PD-L1 blockade | Simlukafusp alfa (FAP-IL2v) + atezolizumab | Basket solid tumor study | Phase II | NCT03386721 ([ClinicalTrials.gov](https://www.clinicaltrials.gov/study/NCT03386721?utm_source=chatgpt.com)) |
| FAP-targeted immunocytokine + PD-1 blockade | Simlukafusp alfa (FAP-IL2v) + pembrolizumab | Advanced/metastatic melanoma | Phase Ib | NCT03875079 ([aacrjournals.org](https://aacrjournals.org/cancerrescommun/article/5/2/358/752055/Phase-Ib-Study-of-Immunocytokine-Simlukafusp-Alfa?utm_source=chatgpt.com)) |
| TGF-β pathway inhibition (anti-fibrotic, stromal/immune exclusion axis) + PD-L1 blockade | Galunisertib (TGF-βR1 inhibitor) + durvalumab | Advanced solid tumors (combination immunotherapy setting) | Phase I | NCT02734160 ([ClinicalTrials.gov](https://clinicaltrials.gov/study/NCT02734160?utm_source=chatgpt.com)) |
| TGF-β pathway inhibition + PD-1 blockade (immune-excluded/MSS settings) | Vactosertib (TGF-βR1 inhibitor) + pembrolizumab | MSS metastatic colorectal cancer / gastric/GEJ cancer | Phase Ib/IIa | NCT03724851 ([ClinicalTrials.gov](https://clinicaltrials.gov/study/NCT03724851?utm_source=chatgpt.com)) |
| ECM remodeling / hyaluronan depletion to improve perfusion and drug/immune access (stromal decompression concept) | Pegvorhyaluronidase alfa (PEGPH20) + gemcitabine/nab-paclitaxel | Metastatic PDAC (HA-high enriched populations in some designs) | Phase III | NCT02715804 ([ClinicalTrials.gov](https://clinicaltrials.gov/study/NCT02715804?utm_source=chatgpt.com)) |
| ECM remodeling (hyaluronan depletion) + ICI | PEGPH20 + avelumab | Advanced solid tumors (chemo-refractory contexts; PDAC-focused designs in some cohorts) | Phase I/II | NCT03481920 ([ClinicalTrials.gov](https://clinicaltrials.gov/study/NCT03481920?utm_source=chatgpt.com)) |
| Repurposed stroma-modifying regimen (angiotensin axis / stromal tension) | Paricalcitol + hydroxychloroquine + losartan (“PHL”) | Pancreatic cancer (stroma-modifying combination) | Noted as interventional clinical study | NCT05365893 ([ClinicalTrials.gov](https://clinicaltrials.gov/study/NCT05365893?utm_source=chatgpt.com)) |
| Losartan-containing combination with multi-agent chemotherapy (stroma-modifying adjunct) | FOLFIRINOX + elraglusib + losartan | Pancreatic adenocarcinoma | Phase I/II | NCT05077800 ([ClinicalTrials.gov](https://clinicaltrials.gov/study/NCT05077800?utm_source=chatgpt.com)) |
